# Phonological similarity affects production of gestures, even in the absence of overt speech

**DOI:** 10.3389/fpsyg.2015.01347

**Published:** 2015-09-09

**Authors:** Nazbanou Nozari, Tilbe Göksun, Sharon L. Thompson-Schill, Anjan Chatterjee

**Affiliations:** ^1^Department of Neurology, School of Medicine, Johns Hopkins UniversityBaltimore, MD, USA; ^2^Department of Cognitive Science, Johns Hopkins UniversityBaltimore, MD, USA; ^3^Department of Psychology, Koc UniversityIstanbul, Turkey; ^4^Department of Psychology, University of PennsylvaniaPhiladelphia, PA, USA; ^5^Department of Neurology, School of Medicine, University of PennsylvaniaPhiladelphia, PA, USA

**Keywords:** gesture, phonological similarity, gesture-language interaction

## Abstract

**Highlights**

- Does phonological similarity affect gesture production in the absence of speech?
- Participants produced gestures from pictures with no words presented or spoken.
- Same pictures and gestures but different training labels were used.
- Phonologically similar labels led to more errors in subsequent gestures.
- Thus, phonological similarity affects gesture production in the absence of speech.

Are manual gestures affected by inner speech? This study tested the hypothesis that phonological form influences gesture by investigating whether phonological similarity between words that describe motion gestures creates interference for production of those gestures in the absence of overt speech. Participants learned to respond to a picture of a bottle by gesturing to open the bottle's cap, and to a picture of long hair by gesturing to twirl the hair. In one condition, the gestures were introduced with phonologically-similar labels “twist” and “twirl” (*similar condition*), while in the other condition, they were introduced with phonologically-dissimilar labels “unscrew” and “twirl” (*dissimilar condition*). During the actual experiment, labels were not produced and participants only gestured by looking at pictures. In both conditions, participants also gestured to a control pair that was used as a baseline. Participants made significantly more errors on gestures in the similar than dissimilar condition after correction for baseline differences. This finding shows the influence of phonology on gesture production in the absence of overt speech and poses new constraints on the locus of the interaction between language and gesture systems.

## Introduction

People spontaneously produce gestures when they talk. In one widely accepted classification system, gestures are divided into four main categories—*deictic gestures* (i.e., pointing to an object, person, or location), *beat gestures* (i.e., quick hand movements highlighting the prosody of the speech without semantic meaning), *iconic gestures*, which represent objects or events such as moving the hand in an arc to refer to direction of an action, and *metaphoric gestures*, which refer to abstract ideas (McNeill, 1992). But gestures need not accompany speech. McNeill (1992) puts gesticulation at one end of the line and sign languages to the other end. As we get closer to sign languages on this continuum, speech diminishes and hands carry more language-like properties. Pantomimes, a class of iconic gestures, fall in the middle of the continuum, and symbolically communicate meaning in the absence of speech.

In this paper, we focus on iconic gestures (specifically pantomimes) that carry meaning and ask: in the absence of speech, is the production of such gestures still affected by linguistic form? Specifically, we probe if phonological overlap between two lexical items bears on the production of gestures that correspond to those lexical items, when overt speech is not produced or required. In answering this question, we address the relationship between language and gesture and the specific properties of a system that gives rise to such cross-modal communication.

Whether speech and gesture form a tightly integrated communication system originating from the same representational system, or whether they are two separate but interrelated systems is a matter of debate (Butterworth and Hadar, 1989; McNeill, 1992, 2005; Alibali et al., 2000; Kita, 2000; Krauss et al., 2000; Goldin-Meadow, 2003; Kita and Özyürek, 2003; Alibali, 2005; De Ruiter, 2007; Hostetter and Alibali, 2008—for detailed reviews of the theories see De Ruiter, 2007; Goldin-Meadow and Alibali, 2013; Pouw et al., 2014). While these accounts differ in various how they view the relationship between language and gesture systems, they all agree that gesture and language are not separate modules with no interactions between them. Some, like McNeill (1992, 2005) posit that the closeness arises from the fact that the two emerge from the same system. Support for this claim comes from studies showing that Broca's aphasics do not necessarily produce gestures to clarify their incomplete speech (Goodglass and Kaplan, 1963; Cicone et al., 1979; McNeill, 1985; Glosser et al., 1986; but see Hadar et al., 1998; Lanyon and Rose, 2009). Others have proposed accounts which view language and gesture as separate, but perhaps interdependent, systems. Of these, some claim that gesture influences language, and not the other way around (e.g., Krauss et al., 2000; see also De Ruiter, 2000 for a lack of influence of language on gestures), while others propose that gesture is indeed influenced by language (e.g., Kita and Özyürek, 2003; Hostetter and Alibali, 2010; see also Hostetter and Alibali, 2008 for a full discussion).

Claims about language influencing gesture production have been tested primarily at the conceptual or morpho-syntactic level, through cross-cultural studies which take advantage of the fact that concepts are expressed differently in various cultures and languages. For instance, when an English speaker expresses a “roll down” event, the one-clause sentence (e.g., he *rolled down*) accompanies a gesture that conflates two types of information: the trajectory of the motion (down) and the manner of the (rolling) (e.g., index finger making circles while moving down). In contrast, Turkish speakers can express the same event in two clauses (e.g., he descended as he rolled) and use two separate gestures (e.g., one for moving down and the other for circular movement) (Kita and Özyürek, 2003; Kita et al., 2007).

Our study also looks at the influence of language over gesture production, but with two main differences from previous studies: (1) We explore the effect in the absence of any overt language produced or required, and (2) we focus on the effect of phonological similarity in language on gesture production, which differs from semantic/syntactic influences targeted in past studies. It is worth emphasizing that this study does not address the origin of gestures (i.e., how they are produced; see Hostetter and Alibali, 2008 for a full discussion). In agreement with most theories, we assume that language and gesture originate from two different systems and interact at some point. Our target question is whether production of gestures is influenced by phonological representations within the language system.

**Figure 1:**
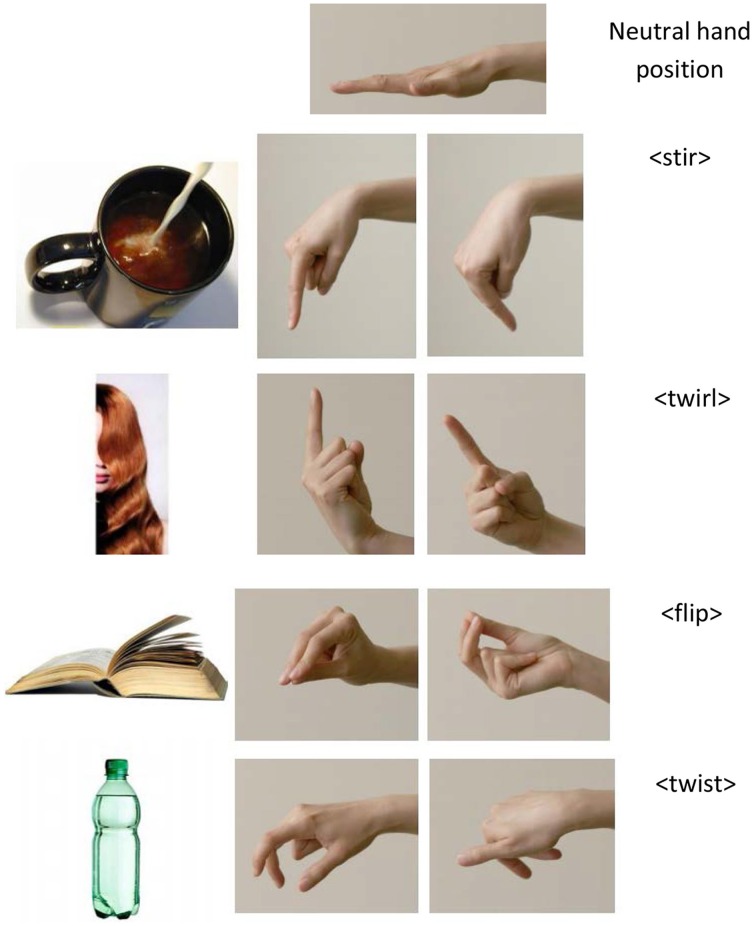
**The experimental materials (pictures) and the target gestures**. The neutral hand gesture is the starting point in all conditions. The gesture immediately adjacent to the pictures of the stimuli portrays the first complete critical hand form that is used for RT coding (before the motion starts). The next picture shows how the motion unfolds.

In a simple paradigm, participants learned to produce four gestures in response to four pictures (Figure 1): (1) <stir> with a cup of coffee with milk pouring into it; (2) <twirl> with a woman's long hair hanging to the side of her face; (3) <flip> with an open book with its pages turning; and (4) <twist> with a bottle with its cap on. All gestures were naturally associated with the pictures, so associations were not arbitrary. Pairing up <twist> and <twirl> created the *experimental pair*. In one condition, these gestures were introduced to the participants with labels “twist” and “twirl.” Because of the high phonological overlap between the two words, this condition was called the *similar condition*. In another condition, the same gestures were introduced as “twist” and “unscrew,” and the condition was called the *dissimilar condition* because of the low phonological overlap between the words in the pair. Participants in both conditions, also gestured to a *control pair* (<stir> and <flip>), the labels of which (“stir” and “flip”) were kept constant across the two conditions. This control pair was motorically similar to the experimental pair (see Methods for details) and was thus useful for capturing any baseline differences between the two conditions that were not caused by the experimental manipulation.

Each experimental and control pair was presented in two phases: (a) a *straight phase*, during which participant produced the gesture corresponding to the picture (e.g., <sitr> for the coffee cup), and (b) a *reversed phase*, during which the participant produced the other gesture (e.g., <flip> for the coffee cup). The reversed phase always followed the straight phase to ensure both items were learned well. The purpose of the reversed phase was to induce more errors, as piloting showed that performance was at ceiling in the straight phase.

## Predictions

**Figure 2:**
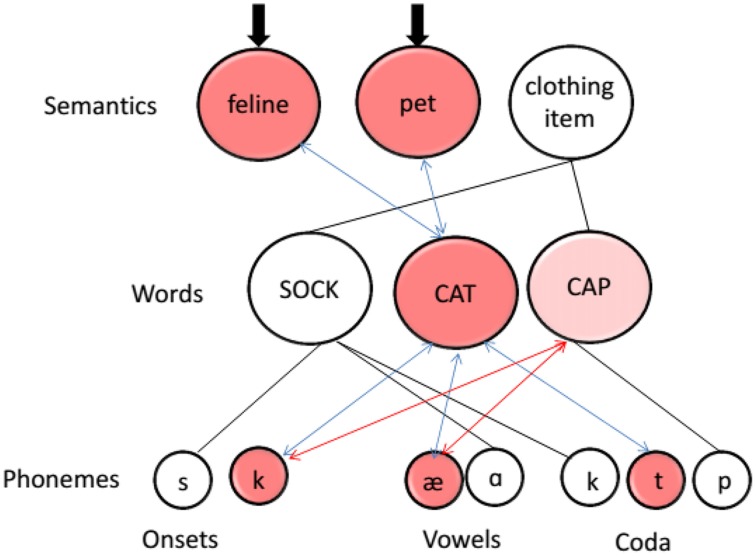
**A schematic diagram of the interactive two-step model of language production (Dell and O'seaghdha, 1992)**. All connections are bi-directional. Blue arrows indicate connections to the target (“cat”). Red arrows indicate connections that activate the phonological competitor (“cap”). Thick black arrows mark input to the semantic layer of the model.

If phonological similarity affects gesture production, we would expect differences in producing gestures for the experimental pair in the similar and dissimilar conditions. Specifically, we would expect the phonological overlap of the two similar labels to interfere with the production of their corresponding gestures. To explain how this would happen, we give a brief review of the architecture of the language production system. Figure 2 is a schematic of the interactive two-step model of language production (e.g., Foygel and Dell, 2000; Nozari et al., 2010; see Dell et al., 2014 for a review). In this model, input to semantic features (e.g., feline, pet, clothing item) activates the abstract word representations connected to those representations (e.g., “cat”). Those words, in turn, activate their phonology clustered according to their position in the word. An important feature of the model is that its connections are bi-directional, meaning that activation not only spreads from higher layers to lower ones, but also backwards from lower layers to higher ones. Therefore, nodes in the word layer not only influence selection of phonemes but are also affected by the activation of phonemes (hence the label “interactive”).

A direct consequence of this interactivity is that words that are not activated in a top-down fashion can become activated via bottom-up connections. In the example given in Figure 2, the model means to retrieve the word “cat.” Semantic features of cat are activated, and the word node “cat” and its phonemes are also activated via top-down connections. Without feedback, there would be no reason for a phonologically similar word, such as “cap,” to also gain activation because it shares no semantic features with “cat” and receives no top-down activation from the semantic layer. However, “cap” can gain activation if there is feedback from phonemes to words. Phonemes /k/ and /æ/, which have become activated through “cat,” feed their activation backwards to other words that share those phonemes, and in this manner activate “cap.” Considerable evidence supports this kind of interactivity in the language production system (e.g., Dell, 1986; Rapp and Goldrick, 2000; Nozari and Dell, 2009; Breining et al., 2015). For our purpose, we adopt the framework and assumptions of the interactive two-step model as they pertain to the word and phoneme layers, namely that similarity at the phoneme layer increases competition at the word layer by activating other word nodes that otherwise would not have been activated. Given that inner speech (i.e., speech that does not become overt) shows a full phonemic structure (Oppenheim and Dell, 2008), the predictions made above should not differ between overt and inner speech.

**Figure 3:**
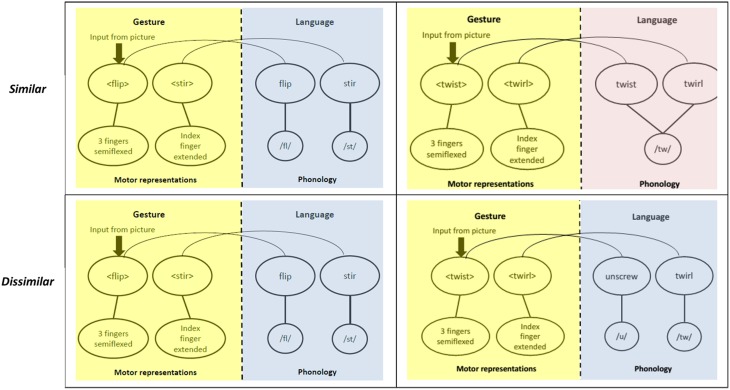
**The rationale of the study**. The first row shows the similar and the second row, the dissimilar condition. The yellow panels show a schema of the process of mapping semantics (from pictures) to motor movements in gestures. The blue and pink panels show a part of the language production system that maps abstract word representations onto their phonology. The three blue panels are similar in that there is no overlap in the word onsets. The pink panel, on the other hand, contains words that share onset phonology.

Figure 3 situates our experimental design in the framework discussed above. The semantic part of the model is not shown, because items in neither pair are semantically related. The figure makes it clear that the gesture parts are identical between the similar- and dissimilar-labels experiments (yellow boxes in the first and second rows). Therefore, in the absence of an influence from the language parts, there should be no differences in performance between the two groups participating in these two experiments. If, however, the language production system is activated during gesture production AND this activation affects the production of gestures, then we would expect different performance in the two experiments. This difference is shown in the blue and pink boxes in Figure 3.

## Methods

### Participants

Sixty right-handed native speakers of English (20 males; Mean age = 20.84 years) participated in the study for either course credit or $10 cash. All participants gave their written consent for participation in accordance with guidelines approved by the IRB committee of the University of Pennsylvania. One participant was excluded because he did not make the proper gestures throughout the experiment, one because he was a native signer of American Sign Language, and three of the participants in the dissimilar condition because they mentioned “twist” in the post-experiment questionnaire (see below).

### Materials

Four motion gestures were targeted: <stir>, <flip>, <twist>, and <twirl>, and were presented in two pairs (Stir-Flip, and Twist-Twirl). Targets were chosen to be motorically similar in the two conditions. Figure 1 shows the four gestures. As can be seen, <stir> and <twirl> both require full extension of the index finger and full flexion of the other four fingers. They also both involve a circular motion. The only difference is that in <stir> the finger is pointing downward, but in <twirl> it is pointing upward. <flip> and <twist> both involve the thumb, the index and the middle fingers in a semi-flexed position, with the other two fingers fully flexed. They also both involve a rotation movement, although this movement requires supination in <flip>, but not in <twist>.

Gestures were elicited by pictures, without presentation of words on individual trials. We, therefore, selected four pictures from Google Images that pilot testing determined corresponded well to each gesture (Figure 1).

### Procedure

Participants were randomly assigned to one of the two conditions. In one condition, the experimental pair was labeled as “twist,” “twirl” during the training phase (similar condition). In the other condition, the same pair was labeled as “unscrew,” “twirl” to break the high phonological similarity between the two words (dissimilar condition). The control pair (labeled “stir” and “flip”) was kept constant across groups. Participants in each condition thus produced gestures on two pairs (the control <Stir-Flip pair>, and the experimental <Twist-Twirl>/<Unscrew-Twirl> pair). The order of presentation of the two pairs was counterbalanced. Each pair was presented in two phases: participants first completed a straight phase, in which they produced the relevant gesture from the picture (e.g., picture = coffee cup 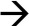 gesture = <stir>). In the reversed phase, when one picture was presented, they had to produce the gesture for the other picture in the pair (e.g., picture = coffee cup 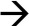 gesture = <flip>). The rationale for including the reversed condition was to elicit enough errors for analysis, as pilot testing showed that the straight task was too simple to elicit errors in most participants. Note that there were only two pictures in each pair, so the target gesture in the reversed phase was unambiguous.

Each session began with training participants on the gestures. For each pair two pictures were presented one at a time, and the experimenter (the second author and an expert in gesture research) first labeled each gesture, and then showed the participant how to perform it. Training was very short, as all four gestures were what participants would have naturally performed. Participants also received training on how to start at a baseline position and come back to it after they completed each gesture. This was a flat hand position (Figure 1) at the level of a marker positioned vertically on the testing table in front of them. Once participants learned to produce the gestures, they started the experiment. At the beginning of each pair and each phase, they first completed six practice trials, three for each gesture. If necessary, they received corrective feedback during practice, and moved on to the experimental phase, during which feedback was no longer provided. On each trial, one of the two pictures appeared on the screen, and a beep was played simultaneously to cue the participant to start producing the gesture. Participants were instructed to produce the target gesture as quickly and as accurately as they could. The picture remained on the screen for two seconds during which a response was expected. ITI was 500 ms during which a fixation cross appeared on the screen. We chose these timings by piloting a different group of participants before the experiment, to ensure that the gap allowed for production of the gesture and returning to the flat baseline before the next trial started.

Each phase for each pair had 16 trials (eight for each gesture presented in randomized order). Participants were allowed to take breaks between each two phases for as long as they wished. In total, each participant completed 64 trials (16 Stir-Flip-Straight, 16 Stir-Flip-Reversed, 16 Twist-Twirl/Unscrew-Twirl-Straight, and 16 Twist-Twirl/Unscrew-twirl- Reversed). After the experiment a questionnaire was administered to participants that asked them if they had considered any other labels for the gestures during the experiment. As the hypothesis of the study relies on the assumption that participants in different conditions thought of labels differently, it was critical to confirm that they did not replace the given labels with different ones on their own.

It is important to keep in mind that each label was only encountered three times during the experiment, once during gesture training produced orally by the experimenter, and once written on the screen at the beginning of each phase of the presentation of a pair (Straight and Reversed). During the trials participants did not see, hear or produce any words.

## Results

### Participant exclusion

In the post-experiment questionnaire, three participants in the dissimilar condition mentioned having thought of <unscrew> as “twist.” These participants were excluded from the analyses. The reason for this exclusion was that the dissimilar condition was only expected to differ from the similar condition if the participant used the unrelated (“unscrew”) label when thinking of the relevant gesture. If, on the other hand, the participant thought of this gesture also with the related label (“twist”), we could not be certain that s/he was using dissimilar labels when making the gestures.

### Error and RT coding

Each session was videotaped using a SONY video camera CCD-TR72. Appendix A in Supplementary Material contains a detailed description of the rules used for coding errors and RTs. We intentionally chose conservative rules to code errors to avoid assigning error codes to possibly correct trials. All trials were double coded by the first and the second author independently (see Appendix A in Supplementary Material for initial checking and subsequent procedures). The coders were blind to the trial labels at the time of coding. They only wrote down the first gesture they saw in the trial. Thus, for many trials, the correct/error status of the trial was only apparent after the coding when the codes were matched with the trials' target labels. Inter-rater reliability was calculated using the Kappa statistic to correct for chance, and was 0.88 (95%CI = 0.84–0.94). The trials for which there was a disagreement were reviewed by both raters and recoded. All trials that were subject to disagreement were then coded by a third rater, blind to the hypothesis of the experiment but trained on the gestures and the coding criteria. If the ratings of the three coders agreed, the code was finalized. If not, that trial was excluded. Only two trials in total were excluded for this reason.

There were a number of ways to code RTs. One was to code the latency from the baseline position to the very first movement. This measure was of limited use. In speech, the equivalent index would be to code the latency of the first sound that triggers the microphone, which might be pre-voicing, lip smacking, or other sounds that do not really mark the beginning of the word of interest. Another, more informative way to code the RTs was to measure the latency of an assumed posture that unequivocally discriminated between the gestures in each pair. These positions are shown in the middle panel of Figure 1, and Appendix A in Supplementary Material contains a full description of the procedures for coding RTs and the advantages of choosing this index for measuring latencies. All data were coded by a trained psychology undergraduate student blind to the hypothesis of the study. Two subjects were chosen at random for each experiment and were double coded independently by the first author. A criterion for reliable coding was set as follows: raters would be considered to have high agreement if they chose the same video frame ±1 (either the frame before or after) as the first unequivocal movement, on 80% or more of the trials. This criterion was met.

## Analyses

### Errors

**Figure 4:**
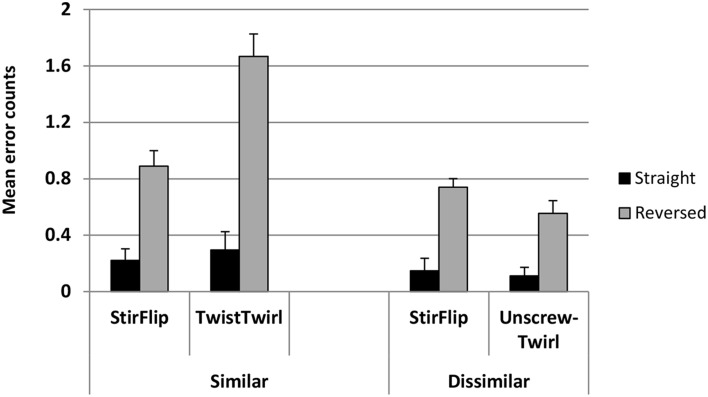
**Mean error counts (+SE) for similar and dissimilar conditions for each pair of words in each phase**.

There were 83 errors made (53 in Twist-Twirl, 30 in Stir-Flip) in the similar and 42 errors made (18 in Unscrew-Twirl, 24 in Stir-Flip) in the dissimilar condition. Figure 4 shows the average error counts (+SE) for the experimental and control pairs in the similar and dissimilar conditions, in each phase.

A multilevel mixed effect regression model was built with accuracy (incorrect or correct) as the dependent variable. Because the dependent variable is binary, a logistic version of the model was employed. Fixed effects included Relation (experimental vs. control pair), Phase (straight or reversed), Condition (similar or dissimilar), their two and three-way interaction terms and order (whether experimental or control pairs were presented first). Random effects included the random intercept of subjects (are some subjects simply more or less error prone in their gesture production?), random slope of Relation over subjects (are some subjects particularly error prone for related or unrelated pairs?), random slope of Phase over subjects (are some subjects particularly error prone in the straight or reversed phases?), and the random slope of Phase × Relation over subjects (are some subjects particularly affected by the combination of relatedness and reversal?). Note that random slopes of Condition and Order are not included, because they are between-subject variables and already appear in the fixed- effect part of the model. There are also no random effects of items in the model because there are only two items in each condition. However, using a mixed-effect model allows us to use the full random effect structure over subjects, which prevents the variance due to these random factors to be ascribed to the fixed effects of interest. Also, a logistic model is the proper statistical choice for categorical independent variables such as errors (for more on mixed effect models and their superiority over ANOVA see Barr et al., 2013; Nozari et al., 2014).

A summary of the model's estimated coefficients, their standard deviations, and their corresponding t statistics and *p*-values is presented in Table 1. Participants made significantly more errors in the more difficult reversed phase collapsed over pairs (*t* = −4.76, *p* < 0.001). They also made more errors on the experimental pair overall, when collapsed across both conditions (*t* = −2.37, *p* = 0.018). The model also showed that participants in the dissimilar condition made in general fewer errors than those in the similar condition (*t* = −3.99, *p* < 0.001). The critical test of our hypothesis was the interaction between Relation and Condition. This interaction tested if there were more errors in the experimental vs. control pair in the similar vs. dissimilar condition, and was significant (*t* = 2.235, *p* = 0.025). Other effects were not significant.

**Table 1:** **Results of the error analysis**.

	**Coefficient**	***SE***	***t***	***p*-value**
**FIXED EFFECTS**				
Intercept	−1.987	0.377	−5.36	< 0.001
Relation	−0.658	0.278	−2.37	0.0178
Phase	−1.997	0.420	−4.759	< 0.001
Condition	−1.309	0.328	−3.986	< 0.001
Order	−0.139	0.214	−0.908	0.364
Relation × Phase	0.483	0.631	0.766	0.444
Relation × Condition	1.007	0.451	2.235	0.025
Phase × Condition	0.133	0.786	0.169	0.866
Relation × Phase × Condition	−0.365	1.084	−0.337	0.736
**RANDOM EFFECTS**				
**Subject intercept**	**Variance**			
Intercept	0.381			
**Subject slopes**				
Relation|Subject	0.143			
Phase|Subject	0.218			
RelationxPhase|Subject	0.0739			

### RT analysis

**Figure 5:**
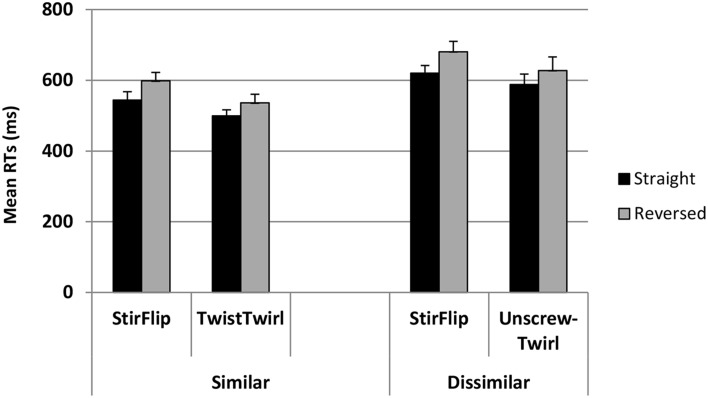
**Mean RTs (+SE) for similar and dissimilar conditions for each pair of words in each phase**.

Error trials were excluded from the RT analysis. Figure 5 shows the average RTs (+SE) for the experimental and control pairs in the similar and dissimilar conditions, in each phase. A multilevel mixed effect regression model, similar to that used in the analysis of accuracy, was employed to analyze the RT data. However, because the dependent variable was continuous, a logistic version was not used. Fixed and random variables and the model structure were the same as in the error analysis model. Results are summarized in Table 2. The model showed that collapsed over both experiments, RTs were significantly faster for the experimental than the control pair (*t* = 3.52, *p* < 0.001), and participants were overall slower in the dissimilar condition (*t* = 4.74, *p* < 0.001). Recall that the error analysis showed that the participants in the dissimilar condition committed fewer errors, hinting at the classic speed-accuracy tradeoff. Order also had a significant effect, such that whichever pair appeared second was responded to more slowly, perhaps due to fatigue (*t* = 2.84, *p* < 0.001). Similar to the error analysis, however, the critical test of the hypothesis was the interaction between Relation and Condition. This interaction was not significant (*t* = −0.99, *p* = 0.322). Neither was the three-way interaction between Relation, Phase, and Condition (*t* = 0.58, *p* = 0.566). Other effects were not significant either.

**Table 2:** **Results of the RT analysis**.

	**Coefficient**	**SE**	**t**	***p*-value**
**FIXED EFFECTS**				
Intercept	59.822	3.138	19.066	< 0.001
Relation	9.23	2.614	3.531	< 0.001
Phase	−3.14	1.894	−1.658	0.102
Condition	8.704	1.835	4.744	< 0.001
Order	3.360	1.183	2.840	< 0.001
Relation × Phase	−2.494	2.667	−0.935	0.353
Relation × Condition	−2.511	2.532	−0.991	0.322
Phase × Condition	4.472	2.289	1.954	0.051
Relation × Phase × Condition	1.857	3.23	0.575	0.566
**RANDOM EFFECTS**				
**Subject intercept**	**Variance**			
Intercept	201.846			
**Subject slopes**				
Relation|Subject	153.380			
Phase|Subject	42.978			
RelationxPhase|Subject	85.745			

In summary, when compared to the control pair (which was identical in the two conditions), participants made significantly more errors on the experimental pair in the similar compared to the dissimilar condition, indicating that the similar labels caused interference. Comparison of the RT patterns revealed no reliable difference between the pattern of latencies in the two conditions, thus refuting the possibility that the specific increase in error rates on the experimental pair in the similar condition was compensated by faster RTs for that specific pair and condition.

## General discussion

We showed that phonological similarity between the lexical labels that could be used to describe motion gestures influences the production of those gestures, even when speech is not produced or required. Participants generated more errors producing conceptually and motorically identical gestures when they thought of them as “twist” and “twirl” rather than “unscrew” and “twirl.” This finding supports theories of gesture production that posit an influence of language on gestures (e.g., Kita and Özyürek, 2003; Hostetter and Alibali, 2008, 2010), and adds to them in two ways. First, no overt speech is required for the linguistic influence to occur. Second, the influence can come from phonological representations that are situated lower than the morpho-syntactic layers in the language system.

Although much work has been dedicated to studying the relation between gestures and language, detailed models of gesture-language overlap that link different levels of representation between the two systems are rare. One prominent exception is the model proposed by Krauss et al. (2000). The model uses the full architecture of Levelt's (1989) feed-forward language production system, and proposes specific points in this architecture as communication ports with the gesture system. The model deserves praise for specifying the most complete representational system for both language and gesture, as well as their interface. As such, it can be used to test specific hypotheses about the locus of interaction between the two systems. For this reason, even though our findings contradict Krauss et al.'s position, we choose to use their model as the starting point, and propose modification to the structure of the model to accommodate our current data. In doing so, we emphasize once again that we are agnostic about the origin of gestures. Pictures may have simultaneously elicited both gestures and their corresponding labels. Alternatively, one may have been elicited before the other or have triggered the other. Finally, gestures may have been produced without their accompanying lexical labels. Our hypothesis and empirical findings address (1) if indeed linguistic labels were activated along with gestures and (2) if the phonological forms of such labels affect the production of gestures.

The claim that language influences gesture production was tested directly in our experiments, and was supported by the observation that having phonologically-overlapping labels increased interference in producing gestures that were otherwise identical. According to Krauss et al.'s (2000) model, the language system can influence the gesture system via the auditory monitor that processes spoken speech. It is noteworthy that the authors do not mean that the form of gestures are affected by language, but simply that a command from the language system “serves as the signal to terminate the gesture.” Perhaps the signal from the articulator in their model does more than just terminate gestures and actually influences the process of gesture production itself. This possibility would account for the fact that gestures were influenced by language in our study. However, there was no speech output in our experiment, and yet the lexical labels clearly influenced gestures. Therefore, the two systems must be linked earlier on, as the linguistic forms never reach the articulator or the auditory monitor. Note that Krauss and his colleagues also discuss cross-modal priming of spatial and lexical representations in “working memory,” but that level seems sufficiently removed from the phonological level to account for phonological similarity effects.

So, at which level of representation does language system communicate with the gesture system? One possibility is the phoneme level. This is appealing for two reasons: (1) our manipulation is phonological, and (2) in Krauss et al.'s (2000) model, gestures influence lexical retrieval when a “kinesic” monitor communicates with the phonological encoder (gesture 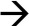 language). The model thus proposes a port of communication for gesture to affect language at the level of the phonological encoder. If such a port is in place, it might be used bidirectionally to also allow language to affect gesture. But there is a drawback to this appealing solution: Regardless of the nature of representations that reach the kinesic monitor, linking the gesture system directly to the phonological encoder is problematic, because it assumes a direct correspondence between gestural features and phonology. This means that there must be a systematic mapping between single phonemes (e.g., /m/) and certain gestural elements. This is unlikely to be true, as it is untrue in language. Conceptual features do not correspond directly to phonemes. For example, the conceptual feature of maleness can be linked to words that begin with a variety of phonemes, such as /f/ in “father” and /m/ in “male.” Same is true for femaleness (/f/ in “female” and /m/ in “mother”). It is exactly this lack of direct correspondence of higher-level representations to their corresponding phonemes that motivates having an intermediate “word” layer in computational models of language production. It is more difficult to define the nature of the gestural representation that maps on to phonemes, as gestures are global and synthetic in nature (McNeill, 1992). They are not simply the sum of their parts, nor do they comply with analytic rules like language units. For this reason, it is even harder to imagine how they could possibly have a one-to-one correspondence with phonemes. For the same reason, it is more reasonable to assume that the gesture system communicates with the language system through the intermediate layer of abstract words, which are more holistic representations themselves, instead of linking directly to phonemes.

Note, however, that a strictly feed-forward language production model such as Levelt (1989) fails to explain the effect of phonological similarity on gestures if it were to link to the language system at levels above phonemes. A higher layer such as the abstract word layer can only reflect phonological influences if it receives signal from the phoneme layer. We therefore propose that our results are most compatible with a model in which an interactive language system with feedback between phonemes and words communicates with a gesture system, most likely at the level of the abstract word forms. It is possible that the receiving end in the gesture system is also a layer of abstract gesture representations, but the need for a mediating abstract representation may not be as obvious as in the language system, because of the possible closer correspondence between the motor features and gesture concepts. Gesture concepts, in so far as such an abstract level is not expressed lexically, are likely to be complex motor programs designed to accomplish a goal, but are still built upon and related to more elementary motor features. The arbitrary mapping between phonological and semantic levels in the language system may not apply to the mapping between motor features and gesture concepts. Regardless, our results would be equally compatible with a model in which word representations link to any representations that are involved in producing motor gestures, abstract or otherwise.

This point addresses a possible question of whether interference induced by similar labels is limited to gestures, or would also be observed in a task in which motor responses are arbitrarily paired with phonologically-similar labels? While the latter is an empirical question, it is important to point out that (1) since motor planning and execution is an integral part of gesture production, finding phonological interference in a task that requires motor planning is neither problematic, nor unexpected given the claims of this paper. (2) There are, however, fundamental differences between tasks that require arbitrary stimulus-response mapping and the task used in this study. Pictures (stimuli) were selected to naturally evoke the related gesture (response), thus the stimulus-response mapping was not critically dependent on linguistic labels. In a task in which response keys are arbitrarily paired with stimulus pictures, there is a much higher chance for using verbal strategies to memorize the correct stimulus-response mapping. As such, demonstration of a phonological effect on motor responses in arbitrary pairing of stimulus-response does not generalize to an influence of phonology on gestures, but discovering the opposite would not be surprising.

One limitation of the study is that the hypothesis was tested on only one pair. This obviously limits our ability to claim that production of all gestures rely on linguistic labels. However, that is not our claim. The study was designed to demonstrate possible, not inevitable, influence of phonology on gesture production, and the demonstration provided here was the proof of concept. Moreover, by including a control pair, we refuted the possibility that some other feature, irrelevant to the critical manipulation, caused the difference, and we corrected differences between conditions using that pair. Future work should focus on examining how various aspects of gestures modulate their sensitivity to linguistic variables. Another objection could be that the difficulty of reversal could have evoked linguistic labels to help participants focus on the correct gesture. The low number of errors in the straight condition alone does not allow independent comparisons, so this possibility cannot be refuted. However, pertinent to our discussion is that (1) the retrieved lexical item was never spoken, and (2) regardless of how the language system was activated, it influenced gesture production in the expected direction (interference in the phonologically-similar condition). Here too, future research must elucidate the conditions in which production of gestures rely more heavily on linguistic labels.

Can the effect be the result of greater difficulty of lexical retrieval from semantics in one case vs. the other? Since imageability was the same between conditions, frequency can be used as an index of the difficulty of lexical retrieval. Words with lower frequency are more difficult to retrieve, so they should be associated with higher error rates (e.g., Kittredge et al., 2008). Log10 frequency of “twist” and “unscrew” are 2.67 vs. 1.39 (Brysbaert and New, 2009), meaning that of the two, “unscrew” is the less frequent. If frequency was driving the effect, we would have expected *more* errors when this label was used, as opposed to “twist”. Finally, “twist” and “twirl” have a higher co-occurrence rate in language than “unscrew” and “twirl.” This higher co-occurrence may indicate a higher similarity in the semantics of the actions defined by the former pair than those defined by the latter. For example, “doctor” and “nurse” co-occur more frequently than “teacher” and “nurse,” because the former pair has a closer semantic link than the latter. However, in the current experiment, the actions and their semantics are identical in the two conditions, thus semantic differences cannot explain the pattern of results. This leaves a second aspect of co-occurrence, namely that of phonological forms. It is possible that words co-occur more frequently because they are phonologically similar (e.g., hanky panky, razzle dazzle). Whether phonological similarity directly affects gesture production or whether it does so through increased co-occurrence of two words, the conclusion remains valid: word-forms, in the absence of semantic change, affect production of gestures.

While our results support an influence of the language system on gesture production, these experiments were not designed to provide empirical evidence to support or refute the influence of the gesture system on language production. We can, however, speculate on this question given the cognitive architecture that our data support. For one, we have shown that an interactive language system is required to explain the effect. Moreover, we have demonstrated that the dynamics of the gesture system are influenced by the language system. While not impossible, it is much less likely that in such an interactive architecture gesture would not influence language production. Empirical evidence from past studies also suggests that it does. For one thing, language production is negatively impacted by eliminating gestures (e.g., Beattie and Coughlan, 1999). Moreover, when language production becomes difficult, speakers gesture more frequently (Chawla and Krauss, 1994; Melinger and Kita, 2007). Also, Frick-Horbury and Guttentag (1998) showed that participants often gesture when in a Tip-of-the-Tongue (TOT) state, and preventing them from gesturing in that state increases the rate of failed lexical retrievals. TOT states arise when the stage of mapping words to phonology is unsuccessful (Dell et al., 1997). The most likely reason is incomplete activation at the word level (Meyer and Bock, 1992). Therefore, extra input from the gesture system to the word level could help resolve the TOTs.

In summary, we provided direct evidence for the influence of phonological forms on gestures. In explaining the results, we arrived at a model that is interactive both within the language system and also between the language and gesture systems. This model is most compatible with evidence that also supports an influence of gesture on language production and even learning (Goldin-Meadow and Butcher, 2003; Iverson and Goldin-Meadow, 2005; Iverson et al., 2008; Rowe and Goldin-Meadow, 2009).

### Conflict of interest statement

The authors declare that the research was conducted in the absence of any commercial or financial relationships that could be construed as a potential conflict of interest.

## References

[B1] Alibali M. W. (2005). Gesture in spatial cognition: expressing, communicating, and thinking about spatial information. Spat. Cogn. Comput. 5, 307–331. 10.1207/s15427633scc0504_2

[B2] Alibali M. W. Kita S. Young A. J. (2000). Gesture and the process of speech production: we think, therefore we gesture. Lang. Cogn. Process. 15, 593–613. 10.1080/01690960075004057119362298

[B3] Barr D. J. Levy R. Scheepers C. Tily H. J. (2013). Random effects structure for confirmatory hypothesis testing: keep it maximal. J. Mem. Lang. 68, 255–278. 10.1016/j.jml.2012.11.001 24403724 PMC3881361

[B4] Beattie G. Coughlan J. (1999). An experimental investigation of the role of iconic gestures in lexical access using the tip-of-the-tongue phenomenon. Br. J. Psychol. 90, 35–56. 10.1348/000712699161251 10085545

[B5] Breining B. Nozari N. Rapp B. (2015). Does segmental overlap help or hurt? Evidence from blocked cyclic naming in spoken and written production. Psychon. Bull. Rev. [Epub ahead of print]. 10.3758/s13423-015-0900-x 26179140 PMC4715795

[B6] Brysbaert M. New B. (2009). Moving beyond Kuèera and Francis: a critical evaluation of current word frequency norms and the introduction of a new and improved word frequency measure for American English. Behav. Res. Methods 41, 977–990. 10.3758/BRM.41.4.977 19897807

[B7] Butterworth B. Hadar U. (1989). Gesture, speech, and computational stages: a reply to McNeill. Psychol. Rev. 96, 168–174. 10.1037/0033-295X.96.1.168 2467319

[B8] Chawla P. Krauss R. M. (1994). Gesture and speech in spontaneous and rehearsed narratives. J. Exp. Soc. Psychol. 30, 580–601. 10.1006/jesp.1994.1027

[B9] Cicone M. Wapner W. Foldi N. Zurif E. Gardner H. (1979). The relation between gesture and language in aphasic communication. Brain Lang. 8, 324–349. 10.1016/0093-934X(79)90060-9 509202

[B10] Dell G. S. (1986). A spreading-activation theory of retrieval in sentence production. Psychol. Rev. 93, 283–321. 10.1037/0033-295X.93.3.283 3749399

[B11] Dell G. S. Nozari N. Oppenheim G. M. (2014). Lexical access: behavioral and computational considerations, in The Oxford Handbook of Language Production, eds Ferreira V. Goldrick M. Miozzo M. (Oxford: Oxford University Press), 88–104.

[B12] Dell G. S. O'seaghdha P. G. (1992). Stages of lexical access in language production. Cognition 42, 287–314. 1582160 10.1016/0010-0277(92)90046-k

[B13] Dell G. S. Schwartz M. F. Martin N. Saffran E. M. Gagnon D. A. (1997). Lexical access in aphasic and nonaphasic speakers. Psychol. Rev. 104, 801. 10.1037/0033-295X.104.4.801 9337631

[B14] De Ruiter J. (2000). The production of gesture and speech, in Language and Gesture, ed McNeill D. (Cambridge: Cambridge University Press), 248–311.

[B15] De Ruiter J. P. (2007). Postcards from the mind: the relationship between speech, imagistic gesture, and thought. Gesture 7, 21–38. 10.1075/gest.7.1.03rui

[B16] Foygel D. Dell G. S. (2000). Models of impaired lexical access in speech production. J. Mem. Lang. 43, 182–216. 10.1006/jmla.2000.271621713062

[B17] Frick-Horbury D. Guttentag R. E. (1998). The effects of restricting hand gesture production on lexical retrieval and free recall. Am. J. Psychol. 111, 43–62. 10.2307/1423536

[B18] Glosser G. Wiener M. Kaplan E. (1986). Communicative gestures in aphasia. Brain Lang. 27, 345–359. 10.1016/0093-934X(86)90024-6 2420412

[B19] Goldin-Meadow S. (2003). Hearing Gesture: How Our Hands Help Us Think. Cambridge, MA: Harvard University Press.

[B20] Goldin-Meadow S. Alibali M. W. (2013). Gesture's role in speaking, learning, and creating language. Annu. Rev. Psychol. 64, 257. 10.1146/annurev-psych-113011-143802 22830562 PMC3642279

[B21] Goldin-Meadow S. Butcher C. (2003). Pointing toward two-word speech in young children, in Pointing: Where Language, Culture, and Cognition Meet, ed Kita S. (Mahwah, NJ: Erlbaum), 85–107.

[B22] Goodglass H. Kaplan E. (1963). Disturbance of gesture and pantomime in aphasia. Brain 86, 703–720. 10.1093/brain/86.4.703 14090524

[B23] Hadar U. Burstein A. Krauss R. Soroker N. (1998). Ideational gestures and speech in brain-damaged subjects. Lang. Cogn. Process. 13, 59–76. 10.1080/0169096983865919570882

[B24] Hostetter A. B. Alibali M. W. (2008). Visible embodiment: gestures as simulated action. Psychon. Bull. Rev. 15, 495–514. 10.3758/PBR.15.3.495 18567247

[B25] Hostetter A. B. Alibali M. W. (2010). Language, gesture, action! A test of the Gesture as Simulated Action framework. J. Mem. Lang. 63, 245–257. 10.1016/j.jml.2010.04.00318567247

[B26] Iverson J. M. Capirci O. Volterra V. Goldin-Meadow S. (2008). Learning to talk in a gesture-rich world: early communication in Italian vs. American children. First Lang. 28, 164–181. 10.1177/0142723707087736 19763226 PMC2744975

[B27] Iverson J. M. Goldin-Meadow S. (2005). Gesture paves the way for language development. Psychol. Sci. 16, 367–371. 10.1111/j.0956-7976.2005.01542.x 15869695

[B28] Kita S. (2000). How representational gestures help speaking, in Language and Gesture, ed McNeill D. (Cambridge: Cambridge University Press), 162–185. 10.1017/CBO9780511620850.011

[B29] Kita S. Özyürek A. (2003). What does cross-linguistic variation in semantic coordination of speech and gesture reveal? Evidence for an interface representation of spatial thinking and speaking. J. Mem. Lang. 48, 16–32. 10.1016/S0749-596X(02)00505-3

[B30] Kita S. Özyürek A. Allen S. Brown A. Furman R. Ishizuka T. (2007). Relations between syntactic encoding and co-speech gestures: implications for a model of speech and gesture production. Lang. Cogn. Process. 22, 1212–1236. 10.1080/01690960701461426

[B31] Kittredge A. K. Dell G. S. Verkuilen J. Schwartz M. F. (2008). Where is the effect of frequency in word production? Insights from aphasic picture-naming errors. Cogn. Neuropsychol. 25, 463–492. 10.1080/0264329070164851 18704797 PMC2963561

[B32] Krauss R. M. Chen Y. Gottesman R. F. (2000). Lexical gestures and lexical access: a process model, in Language and Gesture, ed McNeill D. (New York, NY: Cambridge University Press), 261–283.

[B33] Lanyon L. Rose M. L. (2009). Do the hands have it? The facilitation effects of arm and hand gesture on word retrieval in aphasia. Aphasiology 23, 809–822. 10.1080/02687030802642044

[B34] Levelt W. J. (1989). Speaking: From Intention to Articulation. Cambridge, MA: MITPress.

[B35] McNeill D. (1985). So you think gestures are nonverbal? Psychol. Rev. 92, 350–371. 10.1037/0033-295X.92.3.35019009725

[B36] McNeill D. (1992). Hand and Mind: What Gestures Reveal about Thought. Chicago, IL: University of Chicago Press.

[B37] McNeill D. (2005). Gesture and Thought. Chicago, IL: University Of Chicago Press.

[B38] Melinger A. Kita S. (2007). Conceptualisation load triggers gesture production. Lang. Cogn. Process. 22, 473–500. 10.1080/01690960600696916

[B39] Meyer A. S. Bock K. (1992). The tip-of-the-tongue phenomenon: blocking or partial activation? Mem. Cognit. 20, 715–726. 10.3758/BF03202721 1435274

[B40] Nozari N. Arnold J. E. Thompson-Schill S. L. (2014). The effects of anodal stimulation of the left prefrontal cortex on sentence production. Brain Stimul. 7, 784–792. 10.1016/j.brs.2014.07.035 25129401 PMC4259853

[B41] Nozari N. Dell G. S. (2009). More on lexical bias: how efficient can a “lexical editor” be? J. Mem. Lang. 60, 291–307. 10.1016/j.jml.2008.09.006 20126302 PMC2746698

[B42] Nozari N. Kittredge A. K. Dell G. S. Schwartz M. F. (2010). Naming and repetition in aphasia: steps, routes, and frequency effects. J. Mem. Lang. 63, 541–559. 10.1016/j.jml.2010.08.001 21076661 PMC2976549

[B43] Oppenheim G. M. Dell G. S. (2008). Inner speech slips exhibit lexical bias, but not the phonemic similarity effect. Cognition 106, 528–537. 10.1016/j.cognition.2007.02.006 17407776 PMC2435259

[B44] Pouw W. T. de Nooijer J. A. van Gog T. Zwaan R. A. Paas F. (2014). Toward a more embedded/extended perspective on the cognitive function of gestures. Front. Psychol. 5:359. 10.3389/fpsyg.2014.00359 24795687 PMC4006024

[B45] Rapp B. Goldrick M. (2000). Discreteness and interactivity in spoken word production. Psychol. Rev. 107, 460. 10.1037/0033-295X.107.3.460 10941277

[B46] Rowe M. L. Goldin-Meadow S. (2009). Differences in early gesture explain SES disparities in child vocabulary size at school entry. Science 323, 951–953. 10.1126/science.1167025 19213922 PMC2692106

