# Supplementary material for: Phonological similarity affects production of gestures, even in the absence of overt speech
Source: Front Psychol. 2015 Sep 9;6:1347. doi: 10.3389/fpsyg.2015.01347 (PMC4563879; doi:10.3389/fpsyg.2015.01347)
Supplement: Supplementary file 1 [file DataSheet1.DOCX]

# Appendix A – Coding procedures

## Error coding

The first and the second author collaboratively defined an initial set of criteria for coding errors. The two coders then independently coded the first two subjects according to those rules, and compared the results. Specific issues such as hesitations, not accounted for in the first set, were identified and rules were set on how to handle such cases. The new rules were applied to coding the first two subjects and the rest of the data were then coded independently by the two raters according to the new set of rules. When calculating the inter-rater reliability, the first two subjects were excluded, because the assumption of rater independence no longer held for them. The rest of the subjects were used for calculating Kappa. Below is the final set of rules used to code errors. The rules were chosen conservatively, such that a trial was only coded as an error if there was an obvious incorrect movement.

1. For each trial write down the name of the first gesture you see. If the trial contains two gestures (i.e., a correction), register the first gesture.
2. Write down the name of the gesture only at a point when the gesture reaches the unambiguous first movement of one of the two target gestures. For example, Twirl should be registered when the index finger is extended upward from the flat hand position and other fingers are being flexed. Moving the index finger upward is the opposite of what is required to make the Twist/Unscrew gesture. Hence the upward motion of that finger from the neutral position can be taken as the initial phase of the Twirl motion.
3. If it is impossible by looking at the trial to tell what the gesture is, code it as an error. This is a common case when the generated gesture is a blend of the two target gestures. For example, the subject might perform the Twist motion but with fingers facing up, as in Twirl.
4. Ignore hesitations. Hesitations are defined as motions that are (1) in the direction of the correct gesture, but are halted before completed and then resumed, or (2) unspecified motions of the hand that are not conclusively in the direction of either gesture. These are the equivalent of disfluencies in speech, and are studied in a separate category in language (e.g., Arnold, Nozari, & Thompson-Schill, under review).
5. Ignore omissions or long pauses before beginning. No subject failed at producing a gesture at any given trial. In some cases, the gestures were preceded by long delays. These are dealt with in the RT analysis and are not coded as errors.

## RT coding

When gesture studies use RT coding, they usually code the latency from the resting position to the first hand movement. Upon observing the performance of our participants, we doubted that this measure would faithfully capture the latency to the target gesture on many trials. One reason is because hesitations were not uncommon at the beginning stages of a gesture. This means that the participant initiates a small unspecified hand movement, pauses, and then moves towards the target gesture. This is similar to producing “um… [target word]” in picture naming. Obviously, the latency that marks the beginning of “um” is uninformative about the processes underlying the production of the target word. We therefore devised a different measure that can capture something systematic about the target gestures. This must be a point during the trial that when identified, the observer can make a confident guess about which of the two gestures the subject is going to perform. This should also be a point that can be objectively defined, for the purpose of inter-rater reliability.

After testing various options, we settled on the point where the hand has assumed its preparatory position right before the motion starts. Figure 1 shows this position for each word. For Stir, this is when the index finger is pointing straight down and the other four fingers are flexed. For Flip, this is when the thumb, index and middle fingers are pressed together, and the other two fingers are flexed. For Twist, this is when the thumb, index and middle fingers are semi-flexed in a downward position and the other two fingers are flexed. Finally, for Twirl, it is the opposite of Stir, when the index finger is pointing straight up, with the other four fingers flexed.

To determine the RT for each trial, the rater, a trained psychology undergraduate blind to the hypotheses of the study, viewed each trial in ELAN (European Distributed Corpora Project [EUDICO] Linguistic Annotator), developed by the Max Planck Institute for Psycholinguistics. The program allows for simultaneous viewing of video and sound waveform and precise time-locking. The starting point of the measurement was marked using the onset of the beep on the acoustic wave. To determine the critical point, the coder viewed the video frame by frame. The first frame with the unequivocal posture (see above) was selected. The rater then viewed the subsequent frames one by one and determined the frame where the motion (i.e., stirring, flipping, twisting, or twirling) started. The frame immediately before this frame was chosen to be reference frame. The latency between the beep and this reference frame was coded as the RT for that trial.

This coding method has a couple of advantages:

1. It is not contaminated by unspecified initial motions (i.e., equivalents of fillers in language production).
2. It is sensitive to hesitations. Recall that we ignored hesitations in error coding. However, they may reveal important facts about the ease or difficulty of cognitive processing. This method of coding latencies captures those.
3. While determining the first frame where an unambiguous initial position for the gesture was assumed proved difficult for reliable coding by multiple coders, finding the last frame before the start of the motion yielded very good results in terms of reliability. It is also theoretically motivated, because a pantomime gesture for verbs is only meaningful when it carries motion. A still index finger pointing downwards is likely to be read as a cue for down, below, here, etc. But it is very unlikely to be read as “stir”.
